# Supplementary material for: Supply chain integration and innovation performance of manufacturing firms: The moderating role of research and development investment intensity
Source: PLoS One. 2025 Feb 26;20(2):e0316251. doi: 10.1371/journal.pone.0316251 (PMC11864519; doi:10.1371/journal.pone.0316251)
Supplement: Attachment — Submitted filename: Comment.docx [file pone.0316251.s001.docx]

Here's a breakdown of how to revise your paper for PLOS ONE publication based on the reviewer's comments:

**Abstract:**

Clearly State Aims and Research Gap: Rewrite the abstract to explicitly state the research objectives, the identified gap in current knowledge, and how your study aims to address it. Include brief mentions of your methodology and key findings.

**Literature Review:**

Highlight Significance and Update References: Strengthen your literature review by emphasizing the importance of your research within the existing body of knowledge. Include 3-5 recent references (published in 2024) from PLOS ONE articles that directly address your research topic or methodology. For each reference, incorporate relevant extracts that support your study and showcase the significance of your contribution. Use the PLOS ONE reference style guide for formatting.

**Methodology:**

Explain and Motivate Research Objectives and Methodology: Provide a more detailed explanation of your research objectives and methodology. Motivate your choices by explaining why these specific methods were selected and how they address your research questions.

Deepen Description of Mathematical Model and Variables: Expand on the description of your mathematical model and the variables used. Define them clearly and explain their role in the analysis.

**Results and Discussion:**

Sample Size Information: Include details about your data collection process, particularly the sample size, data collection period, and the rationale for choosing these specific parameters.

Supporting Evidence for Conclusions: Address any assumptions made in your analysis. Explain how your conclusions are supported by your theoretical framework, empirical data, or literature review findings.

**Discussion:**

Deeper Discussion of Implications: Expand on the potential impact of your study. Discuss how decisions across various fields can benefit from the insights gained from your research.

**Conclusion:**

Develop a Dedicated Conclusion Section: Create a separate conclusions section that summarizes your key findings, reiterates your unique contributions (both theoretical and practical), acknowledges limitations of the research, and proposes directions for future studies.

**Final Touches:**

Proofread Thoroughly: Perform a meticulous final proofread to ensure clarity, grammar, and formatting accuracy.

By following these steps and addressing the reviewer's specific concerns, you can significantly improve your paper's chances of successful publication in PLOS ONE.
