# Supplementary material for: Supply chain integration and innovation performance of manufacturing firms: The moderating role of research and development investment intensity
Source: PLoS One. 2025 Feb 26;20(2):e0316251. doi: 10.1371/journal.pone.0316251 (PMC11864519; doi:10.1371/journal.pone.0316251)
Supplement: Attachment — Submitted filename: PLOs ONE review (SC integration).docx [file pone.0316251.s002.docx]

**Reviewer’s comments**

Topic: Research on the Impact of Supply Chain Integration on the Innovation Performance of Manufacturing Enterprises --Based on the Moderating Role of R&D Investment Intensity

**Suggestion:** I suggest the authors should be given an opportunity to improve the study based on my comments provided.

Introduction

The paper empirically examines the impact of supply chain integration on firms' innovation performance through panel data of listed manufacturing companies, and provides an in-depth discussion of the relationship between the two based on the perspective of R&D investment intensity.

**General comments:** The study is relevant because it can added supply chain knowledge especially with evident from developing economy. The study has some potential value but can improved by adhering to the following suggestions.

Topic and introduction

1. I suggest you trimmed the topic to a more precise form. You can remove the “research on…”, “based on,,,” portions. Please refer to other studies in this journal properly shape the topic.
2. Also avoid using abbreviations in the topic.
3. The authors must use more current or recent articles, and properly pitch the gap in extant literature, to bring out the novelty of the study.
4. The gap is not clearly stated. The authors must clearly explain the problem found in extant literature. The gap for both the direct and the moderating relationships must be established. It appears the background information has buried the problem the study aimed to address. So please streamline the background information properly.
5. Also, the authors must cite authoritative statements. For example, in the introduction, the authors stated that “At present, China's economic development model is changing from a crude to an innovation-driven model” but this statement was without any citation. Why? Such deficiencies must be corrected throughout the manuscript.
6. Please convert your gaps into research questions. You can refer to: Aryee, R., Alfa, A. A., Acquah, H., Addey, G. B., & Akoto, E. J. K. (2024). Circular economy, customer citizenship behaviour and firm performance: Some empirical evidence. Business Strategy & Development, 7(2), e377. <https://doi.org/10.1002/bsd2.377>

Theoretical background

1. The section 2 should first capture and describe the theory used in the study. (its concept, variables or assumptions and applicability in the domain). Then it was used to distill or generate the various hypotheses. More importantly, insights from the theory employ (together with literature) should be used to develop the hypotheses. This is somehow lacking in this manuscript and must be corrected.

2. Make sure your in-text citation style (throughout the whole manuscript) is consistent with the standards of the journal

Methodology

1. What is the research design? This was not stated.

2. Did you use primary data or secondary or both? This must be clear in the work.

3. The study’s methodology lacks some key components. The methodology should be properly structured to include research design, research approach, study area, sources and types of data, population, sampling procedure, instruments, data collection procedures, operationalization of variables, data analysis and ethical issues.

4. Concerning the data analysis, it appears the study used the multiple regression analysis technique. Why was this done? Especially when the model has several variables. Such situations (complex models) calls for more robust multivariate analytical technique such as structural equation modeling (SEM). For this is the reason why SEM was developed (see Hair Jr. et al., 2017)….to handle complex models. If possible, please use SEM either the variance or covariance based in your work. Anyway, if you still think the multiple regression is okay, then please provide amble justification for itsusage (instead of SEM) in the manuscript.

5. The results (on beta coefficient, p-values, R^2^ etc.) can be presented in a more acceptable manner, See Aryee et al., (2024). Follow the logical representation this paper.

5. I cannot find the analysis or results of the interaction effects….you can graph the moderating effect to bring more clarity, as was done in Aryee et al., (2024).

Conclusion and discussion

1. Why do you capture conclusion and discussion together. Why not rather have results and discussion together. Then under conclusion you can have sub-sections on implication, limitations and future research directions.

2. Finally, proofread the entire manuscript properly.
