# Supplementary material for: Supply chain integration and innovation performance of manufacturing firms: The moderating role of research and development investment intensity
Source: PLoS One. 2025 Feb 26;20(2):e0316251. doi: 10.1371/journal.pone.0316251 (PMC11864519; doi:10.1371/journal.pone.0316251)
Supplement: Attachment — Submitted filename: Response to Reviewers.docx [file pone.0316251.s003.docx]

**Thank you very much for your suggestions on this article, according to your tips article to make the following changes.**

1. I agree with comments of reviewers. Although, there is a potential in the manuscript but if it gets improved as follows:
   You should improve references.
   The manuscript should be proof read by a language expert to remove grammatical mistakes.

Thank you to the reviewers for their suggestions.

Changes have been made to the article's references as requested by the academic editor. And the grammatical errors in the article have been scrutinized and revised to ensure better quality content.

2. Please review your reference list to ensure that it is complete and correct. If you have cited papers that have been retracted, please include the rationale for doing so in the manuscript text, or remove these references and replace them with relevant current references. Any changes to the reference list should be mentioned in the rebuttal letter that accompanies your revised manuscript. If you need to cite a retracted article, indicate the article’s retracted status in the References list and also include a citation and full reference for the retraction notice.

Thank you to the reviewers for their suggestions.

The reference list of the article has been revised according to the requirements of the journal. Duplicate references are eliminated and the format of the references is modified according to the journal format.

The citation [3] in the references was deleted, so the citation order of the whole film was adjusted. Refer to "Can co-ordination advancement of two-way FDI improve resource misallocation? Evidence from 285," published July 8 in the journal Plos One Format requirements for cities in China and journals. The original quoted content is directly written as the Statistical Bulletin of China's science and technology Investment in 2022,...

The reference citation [43] was deleted. It was found that it was cited twice, and the order of subsequent articles was adjusted.

The cited literature [30] has been modified. Since the citations in the original part are quite old, we have searched for the latest theoretical content and updated this part to the newer literature. The content of the article has also made relevant changes, Change the original text to "Theoretically, resource dependence theory accommodates the simultaneous exploration of the firm's internal environment and external influences, not only elaborating on the importance of internal resources, but also emphasizing the important driving force of the firm's decision-making when subject to external constraints. "is changed to: “Theoretically, resource dependence theory accommodates the simultaneous exploration of the firm's internal environment and external influences, not only explaining the importance of internal organizational coordination, but also emphasizing the interdependent collaboration between firms and their partners.”

At the same time, some older Chinese periodicals have been modified and replaced with newer foreign periodicals. Change citation [52] to [50] Mastio EA, Clegg SR, Pina e Cunha M, Dovey K. Leadership ignoring paradox to maintain inertial order. Journal of Change Management. 2024; 24 (2) : 83-101.

Change citation [63] to [61] Ali S, Muhammad H, Migliori S. R&D investment and SMEs performance: the role of capital structure decisions. EuroMed Journal of Business. 2024.

3. The research topic is interesting. However, the citations are not up-to-date and based on Chinese Journal. In addition, the basic punctuation usage is incorrect, Grammar is not good, and the reference writing has the mistakes. Please revise it before recommending the paper in details. I'd be delighted to review the revised version of the paper.

Thank you to the reviewers for their suggestions.

According to the opinions of reviewers, several old Chinese periodicals are updated to the latest foreign periodicals. The original citation [52] was changed from a Chinese journal to a foreign journal. And according to the new quoted point of view to change the content of the article, beautify the content of the article on the basis of also updated the quoted. the document changes the original "There is inertia in the organization itself" to "Organizations inherently possess inertia, which arises from the inevitable complexity of interdependent structures, routines, and roles.

The older Chinese articles citing [63] are also changed to the latest foreign journals. In terms of the measurement of R&D investment indicators, I checked the latest foreign journals and found that the measurement methods were all the same, so I replaced them with the latest foreign literatures.

Secondly, I carefully checked the grammar and punctuation in the article, and corrected the wrong parts. The sentence of the article is also polished to some extent. The format of the references is also modified using plos one format in endnote software.

4. The manuscript appears technically sound based on the methodology and data analysis conducted. The use of multiple regression models, control variables, and robustness checks suggests that the experiments were conducted rigorously with appropriate replication.

The manuscript is generally written in standard English and is intelligible, with clear organization and logical flow between sections. However, minor grammatical errors and typographical issues were noted, which should be corrected to improve the overall readability.

Thank you to the reviewers for their suggestions.

The article has been carefully checked and revised, some grammatical errors and typographical problems have been solved, and the language of the article has been polished to some extent.
